# Economic impacts of caring for autistic children in Ontario, Canada: report from a pilot study

**DOI:** 10.3389/fpubh.2025.1659801

**Published:** 2025-09-17

**Authors:** Jingjing Xu, Gemma Graziosi, Felipe F. Rodrigues, Renfang Tian, Rachel Birnbaum, Nicole Neil

**Affiliations:** ^1^School of Management, Economics, and Mathematics, King's University College, Western University, London, ON, Canada; ^2^Faculty of Education, Western University, London, ON, Canada; ^3^Department of Statistical and Actuarial Science (DSAS), Western University, London, ON, Canada; ^4^School of Social Work & Childhood and Youth Studies, King’s University College at Western University, London, ON, Canada

**Keywords:** economic impacts, autistic children, autism, costs, families, services

## Abstract

**Introduction:**

Although research on the economic costs of autism is growing, relatively few studies have examined these costs incurred by families of autistic children in Canada.

**Methods:**

This study designed and piloted a survey to capture the broader economic impact of caring for autistic children, including direct and indirect costs. It also sought to gather preliminary data to inform a future full-scale survey and enhance understanding of autism’s economic impact in the Canadian context. The pilot survey was developed through a systematic and iterative process involving a literature review, workshops, and focus group discussions. It was then distributed to families with autistic children in Ontario, Canada’s most populous province.

**Results and discussion:**

A mixed-method analysis of survey responses revealed that financial challenges for these families often begin during the diagnostic process and continue with high out-of-pocket medical and therapy costs. Caregivers also face challenges accessing funding and appropriate support services, contributing to indirect costs such as increased living expenses, childcare, education, and training. Caregivers of autistic children in Ontario experience substantial and multifaceted challenges that are compounded by inadequate public support. Understanding the nature and extent of caregiver expenditures can inform more targeted and efficient policy responses in financial, informational, and practical autism-related support.

## Introduction

Autism spectrum disorder is a neurodevelopmental disability characterized by differences in social communication as well as restricted and repetitive behaviors and interests (1). In Canada, approximately 1 in 50 youth were diagnosed with autism, with males being diagnosed approximately four times more frequently than females (2). When examined by province/territory, Ontario was among the provinces with the highest prevalence rates at 2.1% of its total population.

As the symptoms of autism are typically evident during infancy and childhood and persist throughout adolescence and adulthood, many autistic individuals require varying levels of support across the lifespan. As a result, autism is a disability that can impact caregivers as well as the individual. Caregivers of autistic individuals may experience impacts on their stress and quality of life, financial challenges, and relationship changes (3–7). The increasing recognition of autism highlights the urgent need for accessible, individualized supports that respond to the diverse needs of autistic individuals and their families. At the same time, there is growing concern about the economic impact of caregiving, both in terms of direct costs and broader financial implications for families. Both in terms of direct costs and broader financial implications for families.

Existing estimates of autism-related economic burden suggest that a child’s autism diagnosis has a significant financial impact on their families. For example, regarding direct healthcare costs, Lavelle et al. (8) found that, in the United States, autistic children had higher levels of healthcare office visits and prescription drug use and had higher out-of-pocket healthcare costs overall compared with children without autism. Similarly, a systematic review documented higher average healthcare costs, out-of-pocket costs, and excess costs among autistic children compared to children not diagnosed with a disability and children with other intellectual disabilities (9). In Canada, de Oliveire and Tanner (10) reported higher healthcare-related costs among autistic children compared to children without autism within the areas of inpatient and outpatient care, physician services, prescription drugs, and home care.

In addition to healthcare costs, families with autistic children also encounter significant non-healthcare costs, with some studies reporting that these non-healthcare costs could be much higher than healthcare costs. For example, in Ontario, annual expenses for applied behavior analysis (ABA) therapy are estimated to range between CAD$5,000 and CAD$80,000, depending on the child’s needs (11). Regarding other indirect costs, Lavelle et al. (8) reported that autistic children in the United States have higher non-healthcare costs, particularly in the areas of education and special education. Additionally, loss of productivity experienced by family members poses a major financial impact, resulting in an average annual income loss of approximately US$6,200, accounting for 14% of household incomes (12).

Considering international differences in autism-related policies, the costs associated with autism in Canada likely differ from those in other countries. Therefore, it is crucial to investigate the impacts experienced by families of autistic children and create a comprehensive estimate of the economic impact of autism in Canada, including both direct and indirect costs. However, relatively few studies have examined the economic costs of autism in Canada. Dudley and Emery (13) estimated the lifetime costs associated with care and support for autistic individuals at CAD$1.2 million to CAD$4.7 million, depending on the intensity of support needs. Although this study offers significant insights into the costs associated with autistic adolescents and adults aged 14 to 65, it does not address the distinct financial impacts experienced by families of younger autistic children. These may include direct expenditures related to accessing supports and services and indirect impacts on caregivers’ time, employment, and well-being. McLaughlin and Schneider (14) surveyed Ontario families with autistic children to examine barriers and facilitators to accessing autism services. While their findings highlighted the financial challenges faced by these families, their analysis remains primarily descriptive and is embedded within broader service access issues. A more focused, quantitative investigation of the expenditures caregivers make as a direct or indirect result of their child’s diagnosis could provide a systematic understanding of the economic impacts of an autism diagnosis. Such evidence may also help inform the development of federal and provincial policies to ensure more equitable and efficient allocation of support. This, in turn, will allow for more efficient resource allocation that can potentially reduce long-term costs to autistic children and their families and improve their welfare.

Thus, the goals of this study were two-fold: (1) to develop and trial a survey to guide the selection of appropriate questions for assessing the broader economic impact of an autism diagnosis in children aged 1–18 on their families, capturing both direct and indirect costs, and (2), to collect preliminary data for a future full-scale survey which aims to reach a broader participant base and provide a more comprehensive understanding of the economic impact of autism in Canada. Given that Ontario is Canada’s most populous province and has one of the highest autism prevalence rates among Canadian provinces and territories, and to facilitate community outreach, this pilot study focuses on the province of Ontario.

## Methods

### Study design

We employed a systematic and iterative approach to survey development, which included a comprehensive review of the existing literature on the economic costs associated with families having autistic children, a series of workshops, focus group discussions, and an online pilot survey. This section outlines the key phases of the pilot study.

First, an initial survey version was constructed based on findings from our literature review. Survey items were identified and selected from previously validated instruments and relevant studies, with modifications made to ensure contextual relevance to Ontario (8, 15–20).

Second, we conducted a workshop attended by nine multidisciplinary experts, including researchers from economics, child and youth studies, disability studies, applied behavior analytic intervention, and advocates and professionals working with families with autistic children. The feedback provided by participants was used to help improve the survey’s contextual appropriateness.

Third, a focus group event was organized to include parents of autistic children and gather in-depth qualitative feedback on the survey questions’ clarity, relevance, and comprehensiveness. Based on the comments and suggestions provided by the focus group participants, the survey instrument was further refined.

Fourth, following the in-person focus group discussion, during which researchers were present alongside participants to provide support and clarify questions, an online pilot survey was conducted to assess the feasibility of administering the survey in a self-completed, digital format. While the focus group allowed for in-depth insights within a controlled setting, the online survey was conducted to broaden participation and ensure accessibility for a more diverse sample.

The current survey instrument was guided by the following assumptions to ensure that the information collected accurately reflects the economic impacts experienced by families with autistic children. First, the respondent was the primary caregiver, with knowledge of both caregiving responsibilities and household finances. This was emphasized in the recruitment flyer and survey instructions, with “the primary caregiver of a child diagnosed with autism”, highlighted. Second, the financial impacts were assessed over a 12-month recall period to balance accuracy against potential recall bias. Respondents were instructed to provide their closest estimate, reinforced by prompts, “Please provide the best or closest answer that you can” at both the introduction and the cost-related sections. Third, respondents were assumed to be aware of relevant benefits or support programs they received or were denied (including but not limited to Ontario Autism Program (OAP), Ontario Health Insurance Plan (OHIP), and Canada Child Disability Benefits). The survey included options of these programs and an “other” option to capture additional supports. Fourth, it was assumed that respondents could distinguish expenses and employment changes primarily attributable to the autistic child’s needs. To minimize stigma, questions were framed using inclusive language. For example, “related to your autistic child’s needs” rather than “caused by your autistic child.” Additionally, confidentiality was assured before asking sensitive questions, such as those regarding debt, income, and employment changes. Participants were also given the option to skip any question they were uncomfortable answering without invalidating their participation.

With these assumptions established, the survey included an information sheet, a consent form to inform respondents, and seven sections with closed-ended and open-ended questions. The closed-ended questions covered sociodemographic details of the family and information related to economic costs, such as family out-of-pocket expenditures, special education costs, and changes in parental employment status or working hours due to the child(ren)‘s autism diagnosis. It also included five questions from the GO4KIDDS Brief Adaptive Scale (21). This measure assesses the level of support the child requires, understanding and use of spoken language, and the child’s ability to interact socially with familiar adults and other children. Each item is rated on a 5-point scale with higher scores indicating greater skill level and independence. The open-ended questions provide respondents with opportunities to provide further information about their experiences of caring for an autistic child. The last (seventh) section of the pilot survey covered questions asking for specific and relevant feedback from the respondents regarding the survey design. The first author’s university ethics committee granted ethical approval to conduct focus group discussions and a pilot survey.

### Data collection

#### Focus group

Focus group participants were recruited between May 2024 and June 2024 using snowball sampling through posters to autism support groups and organizations in the London, Ontario area, where the authors are based. Eight caregivers of autistic children, aged 1 to 18, participated in focus group discussions in July 2024. Each caregiver was given a printed version of the survey to complete independently, with the researchers sitting nearby to provide an on-the-spot explanation when necessary. After caregivers completed their survey, they were asked about their perspectives regarding the ease of completing the survey, items they believed were important but missed and their lived experiences.

#### Pilot survey

The pilot survey was conducted online using Qualtrics XM between January 2025 and April 2025. Participants were recruited using snowball sampling and through the Autism Ontario West region’s newsletter. This allowed the research team to reach a broader target population segment without excessively drawing from the potential sample pool for the full-scale survey. We received a total of 74 valid responses from the online survey.^1^ Since we also invited focus group participants to the online survey, we used only the online pilot survey data to report quantitative results.

### Data analysis

We employed a mixed-methods approach to analyze the data, which allowed us to capture both quantitative and qualitative aspects of the focus group discussions and pilot survey findings. During the focus group discussion, participants’ comments and suggestions on the initial survey design and their shared experiences raising an autistic child were recorded. Instead of completing the survey via Qualtrics XM, focus group participants completed a print form, which was then digitally transcribed in August 2024. Comments made during the focus group were used to supplement responses to the pilot survey via annotations, which were also transcribed.

Following the pilot survey closure, data analyses were performed between April 2025 and May 2025, comprising steps of data cleaning, qualitative coding, and statistical validation. Regarding quantitative analyses for the pilot survey data, the sample size was too small to obtain robust regression results. Therefore, summary descriptive data were generated using Stata 18, a widely used statistical analysis software. This provides a preliminary quantitative analysis regarding the included families and children’s characteristics, the economic costs that arose from their children’s autism diagnosis, and the practical and social–emotional impacts commonly experienced by caregivers.

Open-ended survey responses and discussions from focus groups were analyzed inductively using qualitative descriptive analysis to identify recurring patterns, then responses were grouped into meaningful themes derived from the data (22). The first step involved generating descriptive codes from the data through an initial review of all open-ended responses by the second author. These descriptive codes were then sorted into three broad themes: economic impacts, practical impacts, social and emotional impacts. For example, the open-ended response “Early intervention works, yet my son will be 10 years old before we receive any funding from the OAP.” was coded as “Long waitlists for services or funding” and sorted under the theme of economic impacts. The open-ended response “I have had to turn down work projects in order to support my child” was coded as “Adjusting work hours to support child (fewer)” and sorted under the theme of practical impacts. Lastly, the open-ended response “It has also brought us closer as a family, as we have learned to adapt, be patient, and celebrate small milestones” was coded as “Bonding over child’s successes” and sorted under the theme of social and emotional impacts.

The second author conducted the first round of coding using NVivo 15 qualitative data analysis software. The first and second authors then refined the coding framework through several meetings as the inductive coding process progressed. New codes were discussed until a consensus was reached. A second round of coding was then completed by the second author using the finalized coding framework.

### Participants

The valid sample of 74 caregivers had an average age of 36 (standard deviation [SD] = 6.6), and were predominantly married (81.1%), with smaller proportions in common-law or partnered relationships (8.1%), single (5.4%), divorced (2.7%), separated (1.4%), or widowed (1.4%). Most identified as White (73%), followed by Black (15%) and Asian (including South Asian, Chinese, and Southeast Asian backgrounds) (8.1%). The majority (63.5%) had two children, 23% had three, 10.8% had only one child, and 2.7% had more than three. Nearly all respondents (95.9%) were biological parents, with 4.1% identifying as foster, step-, or adoptive parents. Of these 74 caregivers, 64 (86.5%) reported having one autistic child, and 10 (13.5%) reported having two autistic children. No participants reported having three or more autistic children. Therefore, the total number of autistic children in this pilot survey is 84.

Table 1 presents descriptive statistics of key characteristics of autistic children from our online pilot survey sample, including the child’s current age, age at diagnosis, sex, diagnosis, and level of support needs.

**Table 1:** Key characteristics of autistic children.

Variables	*N*	Percentage
Total number of autistic children	84	
Current age		
Below 5	14	16.7%
5–12	48	57.1%
13–18	22	26.2%
Age of Diagnosis		
Below 5	54	64.3%
5–12	28	33.3%
13–18	1	1.2%
Not answered	1	1.2%
Sex		
Male	65	77.4%
Female	19	22.6%
Diagnosis		
Autism only	69	82.1%
ADHD only	4	4.8%
Autism and ADHD	7	8.3%
Autism and Other	3	3.6%
Autism, ADHD, and Other	1	1.2%
Level of support needed		
Requires support for almost all, or most aspects of life	20	23.8%
Requires support for most aspects of life	31	36.9%
Requires support for some aspects of life	21	25.0%
Requires support for only a few aspects of life	8	9.5%
Does not require support	4	4.8%
Use of spoken language		
Speaking	63	75%
Non-Speaking	21	25%
Engage in social interactions with familiar adults		
Shows little or no interest	26	30.9%
Shows limited social interests but will sometimes respond	21	25.0%
Shows some interests, responds to others, but does not initiate social interactions	14	16.7%
Shows clear interest, responds to others, sometimes initiates social interactions	18	21.4%
Engages in a wide range of social interactions	5	6.0%
Engage in social interactions with other children		
Shows little or no interest	28	33.3%
Shows limited social interests but will sometimes respond	18	21.4%
Shows some interests, responds to others, but does not initiate social interactions	22	26.2%
Shows clear interest, responds to others, sometimes initiates social interactions	11	13.1%
Engages in a wide range of social interactions	5	6.0%
Intellectual disability		
Yes	11	13.1%
No	73	86.9%
Other long-term health conditions^a^		
Yes	43	51.2%
No	41	48.8%

^a^Other long-term health conditions include chronic lung condition, asthma, chronic heart disease, diabetes, chronic kidney disease, liver disease (e.g., chronic hepatitis), high blood pressure, a chronic blood disorder, a weakened immune system, Chronic neurological disorder (other than ASD), mental health condition (e.g., depression, anxiety), cancer, arthritis.

## Results

Autism involves range of support needs that impact individuals and their families. Rogge and Janssen (23) conducted a comprehensive review on the economic implications of autism. They identified six main categories of costs: medical and healthcare services, therapeutic costs, special education costs, informal care costs, productivity loss of caregivers, and cost of production loss for autistic adults. Our study, focused on families with autistic children, excluded adult-related costs but captured the other five. Based on data from focus group discussions and the pilot survey, we identified three major themes related to the implications of raising an autistic child: economic, practical, and social–emotional impacts. We presented our findings using descriptive statistics to summarize key quantitative data and descriptive analysis to describe qualitative insights. All monetary values are presented in Canadian dollars unless otherwise specified.

### Theme 1 economic impacts: financial costs and challenges receiving services and funding

Financial costs and challenges accessing services and funding were grouped into the theme “Economic Impacts” because they are closely interconnected challenges that jointly contribute to the overall financial challenges experienced by families.

#### Cost of autism diagnosis

Previous studies on the economic costs of autism typically focus on expenses incurred after the diagnosis. However, findings from our pilot survey highlight that families often face significant financial pressure even before accessing formal supports. In particular, the process of obtaining a diagnostic assessment can result in notable out-of-pocket expenses. While 67.9% of caregivers reported that the cost of an autism diagnosis was fully covered by either a public provincial health plan or private insurance, 19% had to pay partially out-of-pocket, and 10.7% bore the full cost themselves. For those who paid partially, the average out-of-pocket expense was $2,205, with a wide variation (SD = $2,654), ranging from $75 to as much as $5,000. Families who paid the entire cost out-of-pocket reported an average expense of $2,850 (SD = $1,693), with payments ranging between $200 and $10,000.

#### Challenges receiving an autism diagnosis

In Ontario, autism assessments conducted by qualified medical professionals are publicly funded through the Ontario Health Insurance Plan (OHIP), meaning that there is no cost to eligible Ontario residents. However, according to a report by Autism Ontario in 2019, families need to wait from several months to over a year for the diagnosis of autism.^2^ As a result, many families seek assessments from private providers and incur substantial out-of-pocket expenses.^3^ In Ontario, autism assessment from private providers can cost between $1,000 and $5,000. To obtain a timely assessment for their child(ren), some caregivers even pursued assessments outside Ontario or internationally, incurring additional travel and accommodation costs that add to the already high assessment expenses: “Because of the waitlist we traveled outside of the country and got a diagnosis in India; that was immediate access to a doctor but cost about $10,000. We wanted to do it as she is my only child, I want her to get help as early as possible”.

In addition to diagnosis costs (34.5%) and long waitlists (15.5%) acting as barriers to families receiving an autism diagnosis, participants reported experiencing delays due to difficulty accessing service providers in their area (21.4%), not being aware of where to seek professional help (14%), concerns about the stigma associated with their child receiving an autism diagnosis (22.6%), their initial concern not seeming to relate to autism (36.9%), and a lack of awareness regarding autism to begin with (45.2%).

#### Out-of-pocket autism-related medical and therapy costs

Table 2 reports the out-of-pocket costs incurred for autism-related medical care and services and therapies and intervention treatment in the past 12 months, conditional on positive out-of-pocket expenses. Zero out-of-pocket expenses are excluded in Table 2 to highlight the level of financial costs among those who incurred costs.

**Table 2:** Out-of-pocket costs for autism-related medical care, therapies and intervention treatment in the past 12 months (in Canadian dollars).

Types of Costs	Mean	Standard Deviation	Median	Min	Max	95% Confidence Interval^c^	*N* ^d^
Autism-related medical visits/appointments	$4,350	$15,565	$500	$4	$80,000	[$1,013, $14,631]	26
Applied behavior analysis (ABA)/behavior therapy	$3,129	$4,772	$1,000	$30	$16,000	[$894, $6,072]	13
Language therapy	$1,837	$2,774	$700	$5	$10,000	[$636, $3,400]	15
Occupational therapy	$1,103	$1,032	$885	$150	$2,500	[$207, $2,000]	6
Physiotherapy	$1,442	$1,806	$750	$150	$5,000	[$467, $2,942]	6
Mental health services^a^	$591 ($10,541)	$489 ($34,473)	$500 ($500)	$250 ($250)	$2,000 ($120,000)	[$386, $909] ([$406, $34,614])	11 (12)
Respite care	$476	$484	$252	$200	$1,200	[200, 1,200]^e^	4
Social skill training	$1,098	$1,053	$715	$10	$3,000	[$608, $1,637]	16
Vocational/job skill training	$1,400	$1,039	$2,000	$200	$2,000	[$200, $2,000]^e^	3
Total medical/therapy costs^b^	$7,538	$2,1,456	$1,500	$8	$122,000	[$3,349, $17,401]	46

^a^One respondent reported a mental health service cost of $120,000. Since this represents an outlier, we presented two sets of summary statistics. The main results exclude this extreme value, while the values in parentheses include it.

^b^Total medical and therapy costs were calculated by summing all reported costs across categories for each respondent. Since caregivers incurred different combinations of medical and therapy costs, the reported mean or median total medical and therapy costs are not equivalent to the sum of the means or median values for each individual cost category. The same applies to all other summary statistics of total medical and therapy costs.

^c^Given the small sample size and potential deviation from normality, we computed 95% confidence intervals (CIs) for the means using a non-parametric bootstrap, where 10,000 bootstrap samples are created by resampling with replacement from the original sample. This approach does not assume a specific data distribution and provides a more robust uncertainty estimation than the traditional CIs (omitted from the table for readability, available upon request), which in some cases yield implausible negative lower bounds, even though all observed data are positive.

^d^Since participants did not incur all types of costs, the number of responses (N) varies by cost category, reflecting only those who reported each specific type of expense.

^e^The bootstrap CIs is identical to the sample’s minimum and maximum values, this is because with very few data point (n ≤ 5), the bootstrap resample process cannot generate enough variability to estimate a meaningful distribution.

Previous studies have documented that a child’s support needs are positively associated with the amount of incurred costs (13, 23). To investigate whether caregivers in our survey report similar patterns, we summarized total out-of-pocket autism-related medical and therapy costs by the level of the child’s needs in Table 3. While formal statistical testing was not feasible due to sample size limitations, Table 3 shows descriptive trends of increasing costs with higher support needs. We observed that, median reported costs were numerically higher among children with greater support needs, accompanied by greater cost dispersion (maximum $1,900–$7,500 for children requiring lower level of support vs. maximum $80,000–$122,000 for those requiring higher levels of support). However, the bootstrap-derived 95% confidence intervals (CIs) for mean costs varied across children’s need levels without demonstrating a consistent directional trend. Although cost uncertainty was widest for children require support for most aspects of life ($2,938–$18,466), those requiring support for only a few aspects of life exhibited wider intervals ($756–$5,180) than children requiring support in almost all aspects of life ($1,205–$4,316). This unexpected pattern may partly be attributable to small sample size, it suggests complex relationships between support needs and costs that require further investigation in larger, stratified samples. Therefore, these results should not be interpreted as demonstrating definitive associations but rather highlight the need for future research with larger sample to better characterize the cost-support association.

**Table 3:** Total out-of-pocket costs for autism-related medical care, therapies and intervention treatment in the past 12 months by level of child’s needs (in Canadian dollars).

Level of support needs	Mean	Standard Deviation	Median	Min	Max	95% Confidence Interval^b^	*N* ^c^
Requires support for almost all aspects of life	$2,546	$3,127	$1,650	$225	$11,145	[$1,205, $4,316]	14
Requires support for most aspects of life^a^	$9,269 ($14,637)	$18,628 ($30,574)	$2,050 ($2,200)	$115 ($115)	$80,000 ($122,000)	[$2,938, $18,466] ([$3,995, $25,974])	20 (21)
Requires support for some aspects of life	$1,453	$1,527	$938	$175	$4,600	[$634, $2,440]	10
Requires support for only a few aspects of life	$2,578	$2,904	$1,200	$500	$7,500	[$756, $5,180]	5
Does not require support	$1,413	$535	$1,500	$840	$1,900	[$840, $1,900]^d^	3

^a^Again, since one caregiver in this category reported an exceptionally high level of costs, we present two sets of summary statistics, one including this outlier in the parentheses and one excluding it.

^b^Given the small sample size and potential deviation from normality, we computed 95% confidence intervals (CIs) for the means using a non-parametric bootstrap, where 10,000 bootstrap samples are created by resampling with replacement from the original sample. This approach does not assume a specific data distribution and provides a more robust uncertainty estimation than the traditional CIs (omitted from the table for readability, available upon request), which in some cases yield implausible negative lower bounds, even though all observed data are positive.

^c^The Numbers reported here differ from those in Table 1, which presents the key characteristics of the autistic children. This discrepancy arises because some caregivers did not report any ASD-related medical or therapy costs. We provide potential explanations in the following section for this observation regarding the non-occurrence of autism-related medical and therapy costs.

^d^The bootstrap CIs is identical to the sample’s minimum and maximum values, this is because with very few data point (n ≤ 5), the bootstrap resample process cannot generate enough variability to estimate a meaningful distribution.

#### Challenges receiving funding and autism-related medical and therapeutic services

A majority of caregivers (80%, 59 out of 74) reported actively seeking government funding after their child(ren) ‘s diagnosis. Among them, 52.5% received either partial or full funding, 34% are still waiting for funding, and 12.5% reported being rejected. Additionally, the process often involved significant delays for those who received government funding. The average waiting time was 1.5 years, and 35% of children either waited or are still waiting for more than 1 year to receive funding. More than one caregiver shared that their family waited 5 years to access funding for their child. As explained by one caregiver:

Although we are just beginning our journey of navigating the Ontario Autism Program, we have already experienced and heavily feel the impact that less funding to a vital program has. Lack of funding puts all the financial burden on the families and puts us in the position of spending thousands of dollars a year on critical services, or watching our children get left behind, abandoned.

Table 4 presents summary statistics on total autism-related medical and treatment costs in the past 12 months based on whether families received funding. We included the reported zero values in Table 4 to reflect the broader distribution of out-of-pocket payments and to illustrate how funding status was associated with these costs. While the small sample size limited the generalizability of the results, the descriptive data suggest that families without funding tended to report higher out-of-pocket payments than those receiving full or partial funding, with maximum out-of-pocket expenditures exceeding those of funded families by more than tenfold. The bootstrap confidence intervals also indicate that families without funding had wider variability in out-of-pocket costs. While these differences may indicate that funding status is associated with the magnitude and variability of out-of-pocket costs, they should be interpreted cautiously as exploratory patterns that require confirmation in larger, stratified samples.

**Table 4:** Total out-of-pocket autism-related medical and therapy costs in the past 12 months by funding status (in Canadian Dollars).

Funding status	Mean	Standard deviation	Median	Min	Max	95% Confidence interval^b^	*N*
Received full amount of requested funding	$1,511	$2,261	$250	$0	$7,500	[$322, $2,829]	18
Received partial funding	$1,572	$2,392	$363	$0	$7,000	[$315, $2,789]	11
Do not receive funding^a^	$6,741 ($10,857)	$16,426 ($27,098)	$1,500 ($1,500)	$0 ($0)	$80,000 ($122,000)	[$3,244, $21,842] ([$4,770, $29,037])	27 (28)

^a^These include families who are waiting for funding, have not yet received it, or those who were rejected. Since the parent who reported paying $12,000 for mental health care did not receive funding, and this large amount is considered an outlier, we again provide two sets of summary statistics, one including the outlier and the values in parenthesis excluding it.

^b^Given the small sample size and potential deviation from normality, we computed 95% confidence intervals (CIs) for the means using a non-parametric bootstrap, where 10,000 bootstrap samples are created by resampling with replacement from the original sample. This approach does not assume a specific data distribution and provides a more robust uncertainty estimation than the traditional CIs (omitted from the table for readability, available upon request), which in some cases yield implausible negative lower bounds, even though all observed data are positive.

Notably, in addition to reporting zero out-of-pocket expenses, some caregivers reported no costs in the past 12 months. This did not necessarily reflect an absence of support needs. In open-ended responses, several caregivers explained that their lack of spending was due to financial constraints, not by choice. As members of lower-income households, they were unable to afford services related to their child’s support and were still waiting for government-funded programs to begin.

Additionally, 26% of the caregivers (22 out of 74) reported that in their opinion, their child(ren) needed medical care, services, therapies or intervention supports related to their autism but did not receive them in the past 12 months. The top three barriers included being unable to take time off to visit a health professional (21.3%), the cost of services (18.6%), and uncertainty about the child’s needs (16.0%). 10.6% of caregivers reported that their child(ren) are currently on a waitlist, and 12.0% indicated difficulty securing a regular healthcare provider or obtaining a referral for services. Regarding early intervention services, the high cost (25%) and lack of available service providers (10.4%) prevented caregivers from enrolling their children for the required hours. Therefore, the non-occurrence of autism-related costs in the past 12 months could also be due to the child(ren) still waiting for the service. As one caregiver shared: “Regrettably, my child was egregiously denied access to crucial medical treatments, therapies, and interventions for their autism needs over the past 12 months, resulting in significant delays in their development and well-being.”

Moreover, in our survey, participants were asked whether they would prefer better public sector services or more money given directly to families. Caregivers indicated similar interest in both better services (40%) and direct funding for families (60%). Caregivers who indicated they would prefer direct funding said that receiving money directly would grant them more flexibility in deciding what services their child can receive due to affordability. Alternatively, caregivers who advocated for specific service improvements emphasized the need for improving the quantity and quality of services via increased funding, better organization to aid families in locating services, more equitable and consistent service access, and shorter wait times. One caregiver’s experience exemplified this concern: “My son knows that he is not being included in extra-curricular activities like his brothers because he is different from other kids his age. More service funding is needed so he can participate fully.”

#### Extra costs as a direct result of child’s autism diagnosis

In addition to medical services and treatment, families also incurred various ancillary expenses as a direct result of supporting their child(ren) ‘s autism-related needs. Following Roddy and O'Neill (20), we categorized these extra expenses into 10 groups, as shown in Table 5. For living expenses, examples such as special diet, special clothing, repairing damage, extra heat, extra electricity, and extra laundry, were listed to guide responses. Participants were asked to provide specific types of living expenses in an open-ended response box. Since different types of extra costs may occur at varying frequencies, respondents were given the flexibility to report these costs on a weekly, monthly, or yearly basis, to facilitate accurate reporting. The costs in Table 5 are presented on a yearly basis. For responses provided on a weekly basis, yearly costs were estimated by multiplying weekly expenses by 52, assuming 52 weeks in a year. Monthly costs were converted to yearly, multiplying the reported monthly amount by 12.

**Table 5:** Extra costs directly related to supporting the autistic child in the past 12 months (in Canadian dollars).

Types of costs	Mean	Standard deviation	Median	Min	Max	95% Confidence interval^b^	*N*
Living expenses	$26,310	$68,389	$4,800	$50	$432,000	[$12,928, $48,815]	55
Childcare costs							
During school year	$10,669	$11,689	$5,600	$600	$41,600	[$5,975, $16,100]	20
During holidays	$22,747	$31,260	$6,100	$120	$108,000	[$10,468, $37,364]	20
Respite care	$2,340	$1,845	$1,950	$240	$5,000	[$653, $3,520]	6
Autism-friendly activities	$1,273	$1,463	$900	$25	$5,000	[$736, $1,901]	22
Travel costs	$3,604	$5,980	$1,440	$100	$24,000	[$1,391, $6,641]	19
Training costs	$ 15,517	$20,399	$5,400	$100	$70,000	[$8,434, $28,460]	16
Technology costs	$6,907	$19,126	$1,000	$55	$98,000	[$1,825, $15,379]	27
Therapy equipment	$3,522	$6,717	$1,000	$100	$28,800	[$1,254, $6,954]	19
Other expenses	$5,108	$4,087	$3,640	$2,000	$12,000	[$2,200, $9,213]	5
Total extra costs^a^	$39,325	$83,740	$8,390	$50	$499,200	[$24,568, $63,611]	72
New debt	$17,137	$17,468	$12,000	$350	$65,000	[$10,645, $24,240]	26

^a^Total extra costs are calculated by summing each respondent’s reported costs across categories. Since caregivers incur different combinations of extra costs, the reported mean or median total extra costs are not equivalent to the sum of the means or median values for each cost category. The same applies to all other summary statistics of total extra costs.

^b^Given the small sample size and potential deviation from normality, we computed 95% confidence intervals (CIs) for the means using a non-parametric bootstrap, where 10,000 bootstrap samples are created by resampling with replacement from the original sample. This approach does not assume a specific data distribution and provides a more robust uncertainty estimation than the traditional CIs (omitted from the table for readability, available upon request), which in some cases yield implausible negative lower bounds, even though all observed data are positive.

Table 5 highlights that the largest extra costs reported by caregivers, based on median values, were living expenses, childcare (both during school terms and holidays), and training costs, while spending on respite care appeared to be the lowest among the extra costs. Though conducted in different countries, this pattern is similar to the findings reported by Roddy and O'Neill (20) in Ireland.

The second-to-last row in Table 5 presents the total annual extra costs directly related to supporting the autistic child over the past 12 months. Almost all caregivers (72 out of 74) reported incurring costs beyond medical and therapy expenses for their autistic child(ren). These additional costs ranged from $50 to $499,200 annually, with a median of $8,390. This is more than four times the median annual medical and therapy cost of $1,500 reported in Table 2. This finding is similar to previous literature which documents a higher non-medical cost compared to medical/therapy-related expenses (see, for example, (17) for the UK; (8) for the US; (20) for Ireland).

To further explore how these costs varied by the level of a child’s needs, Table 6 presents summary statistics of total extra expenses conditional on a child’s support needs. Although the small size constrained the strength of statistical inference, the observed distribution of these costs suggested that families with children of higher level of needs reported greater extra expenses. Cost uncertainty, as reflected in the bootstrap-derived confidence intervals, were also larger and wider for children requiring support in almost all aspects of life ($19,887, $104,077) or most aspects ($24,820, $98,582) compared to those require some support ($5,940, $19,535), only a few support ($5,744, $72,394), or no support ($7,935, $13,045), indicating greater variability in higher-need groups. Again, these results should be interpreted as descriptive patterns rather than evidence of an association, and confirmation in larger, stratified samples is needed.

**Table 6:** Total extra costs directly related to supporting the autistic child in the past 12 months by level of child’s needs (in Canadian dollars).

Level of child’s needs	Mean	Standard deviation	Median	Min	Max	95% Confidence interval^b^	*N*
Requires support for almost all aspects of life	$56,483	$100,078	$11,040	$1,774	$ 376,800	[$19,887, $104,077]	20
Requires support for most aspects of life	$50,679	$100,176	$10,080	$160	$499,200	[$24,820, $98,582]	27
Requires support for some aspects of life	$12,114	$16,500	$4,240	$50	$64,600	[$5,940, $19,535]	21
Requires support for only a few aspects of life	$33,031	$54,877	$9,500	$700	$160,200	[$5,744, $72,394]	8
Does not require support ^a^	$10,490 ($8,580)	$3,116 (N/A)	$10,290 ($8,580)	$7,290 ($8,580)	$14,090 ($8,580)	[$7,935, $13,045] (N/A)	4 (1)

^a^Three children in this category come from families with two children diagnosed with autism. While these three children were reported as not requiring support, their siblings with autism were reported requiring support in some or most aspects of life. Since it is difficult to separate certain expenses such as extra laundry or transportation costs between the two children, the cost statistics reported may not accurately reflect the expenses incurred for children who do not require support and may therefore be overstated. To address this, we also report separate statistics in parentheses, reflecting the extra costs for families with only one autistic child who does not require support.

^b^Given the small sample size and potential deviation from normality, we computed 95% confidence intervals (CIs) for the means using a non-parametric bootstrap, where 10,000 bootstrap samples are created by resampling with replacement from the original sample. This approach does not assume a specific data distribution and provides a more robust uncertainty estimation than the traditional CIs (omitted from the table for readability, available upon request), which in some cases yield implausible negative lower bounds, even though all observed data are positive.

In addition to incurring extra costs, 36% of caregivers reported that there were expenses that they either were not able to afford or did not have sufficient money to meet their autistic child(ren) ‘s needs. These included expenses for additional therapy sessions such as ABA, speech therapy, medications, specialized education and childcare, respite care, transportation, groceries, housing, and damage repairs. Moreover, as shown in Table 5, 39% of caregivers reported having gone into debt within the last year to cover expenses related to supporting their child’s needs, with amounts ranging from $350 to $65,000. Additionally, 45.2% reported receiving financial support from external sources (e.g., family, friends, and support groups) to help make ends meet.

Regarding childcare services in particular, caregivers reported being unable to access services not only due to high cost, but also due to failing to meet eligibility requirements (25%), being located too far away geographically (25%), and/or experiencing long waitlists (62.5%). Some caregivers mentioned that providers were not willing to accommodate their child due to extensive support needs, with one caregiver stating that their child was “kicked out of their daycare program.”

On the other hand, while caregivers incurred a significant amount of extra costs to meet the special needs of their autistic child, many also mentioned cutting back on expenses that they considered optional to afford the more essential expenses. These optional expenses included entertainment and other recreational activities, vacations, television, personal vehicles, clothing, certain grocery items, and investments in savings accounts. As one caregiver expressed in the open-ended responses: “Autism’s crushing financial burden has forced us to sacrifice financial security, homeownership, and retirement savings to cover therapy, medical expenses, and specialized care.”

### Theme 2 practical impacts: employment, housing, and schooling

#### Employment changes

55.4% of caregivers reported that their child(ren) ‘s autism diagnosis has affected the employment status of their household. Similar to those discussed in previous studies (e.g., (24, 25)), a child’s autism diagnosis would result in caregivers working more or fewer hours. On the one hand, caregivers may increase their working hours to cover additional financial costs associated with their child(ren)‘s treatment, therapies and other related expenses. On the other hand, some caregivers are forced to reduce their working hours or even leave employment to take care of their autistic child(ren) and support their special needs. In our pilot sample, 70% reported a reduction in working hours, while 30% reported an increase in working hours. Open-ended responses supported these impacts. One caregiver noted: “We have given up one parent’s career. Therefore, her salary would have been $110,000 before taxes. Plus, we are out the costs of homeschooling as the education board is unable to accommodate our child’s behaviors,” while another parent shared: “… We need to work much longer than expected… we purchased a house that our son with autism could live in after we are gone with care support as needed.”

It should be noted that, even if their regular working hours were not affected, 72.6% (*N* = 65) of respondents reported that they, their partners, or extended family members required time off to support their autistic children. On average, caregivers took 15 days off work, with a standard deviation of 10 days. Table 7 summarizes changes in caregivers’ employment status due to their child(ren) ‘s autism diagnosis.

**Table 7:** Changes in employment status for caregivers.

Average household income and changes in weekly working hour	*N*	Percentage
Average household income before child’s autism diagnosis		
Less than 25,000	5	6.8%
25,000-100,000	24	32.4%
100,000-200,000	35	47.3%
Above 200,000	6	8.1%
Unknown	4	5.4%
Average household income in the last 12 months		
Less than 25,000	4	5.4%
25,000–100,000	30	40.6%
100,000–200,000	37	50%
Above 200,000	2	2.7%
Unknown	1	1.3%
Increase in weekly working hours due to child’s autism diagnosis	13	
Less than 7 h	1	7.7%
7–14 h	5	38.4%
15–21 h	2	15.4%
22–28 h	3	23.1%
29–35 h	1	7.7%
More than 35 h	1	7.7%
Decrease in weekly working hours due to child’s autism diagnosis	28	
Less than 7 h	6	21.4%
7–14 h	4	14.3%
15–21 h	9	32.1%
22–28 h	4	14.3%
29–35 h	0	0%
More than 35 h	5	17.9%

#### Housing changes

As mentioned in the Extra Costs section, some caregivers experienced large expenses related to housing, including rent, utilities, home renovations, and damage repairs. Among caregivers, 27.4% reported spending between $2,000 and $5,000 in the past 12 months, while 17.8% reported spending between $5,000 and $10,000 on house improvements or modifications to meet the unique needs of their children. Specifically, these expenditures included adding soundproofing to rooms, installing sensory equipment/furniture, redoing floors, making rooms larger, and installing “childproofing” mechanisms. Although these modifications were aimed at improving the child’s well-being, the magnitude of the changes that were made can take a toll on some parents, with one stating in the open-ended response that “Sadly, our once warm and welcoming home has been drastically altered, its character erased by the numerous modifications necessitated by my child’s autism, including padded walls, restrictive gates, and reinforced windows, all stark reminders of the suffocating grip of this disorder.”

Additionally, among the 19 caregivers who reported moving to a new home after their child(ren) ‘s autism diagnosis, 63.2% indicated that the relocation was undertaken to meet their child’s needs better. Reasons mentioned for this included cheaper living costs allowing them to afford other essential expenses, being closer to a higher quantity and quality of support services and treatment opportunities, being closer to better employment opportunities, and greater stability in living accommodations.

#### Schooling

A portion of caregivers (12.3%) indicated they experienced major changes in conditions related to their child’s schooling. Regarding aide and education support at school, while 21% of caregivers reported their child(ren) received no in-class support, a majority indicated their child(ren) received either dedicated fulltime education support (50%) or shared education support (29%). However, experiences varied significantly between caregivers. In the open-ended responses, some caregivers reported that the school provided their child with various accommodations and/or modifications to help meet their needs, including listening and communication devices, furniture and mobility aids, sensory equipment, and transportation. One caregiver acknowledged how helpful these resources provided by the school were for their child: “My child relies heavily on their AAC device, adaptive PE equipment, and wheelchair accessible transportation, which have been thoughtfully provided by their school care provider, significantly enhancing their educational experience and overall well-being.” In contrast, some caregivers felt that the support provided by the school was inadequate in meeting their child’s needs. For example, one caregiver mentioned how they had to move their child “to a school that was willing to follow the IEP.” Indeed, depending on the type of formal diagnosis a child has, the amount of support schools are willing to provide varies. One parent reported that the school “would not provide my child with any support because his diagnosis was no intellectual or verbal impairment.” As a result, the child fell behind in several areas of their education, increasing their need for further services in the future and increasing the burden on caregivers to afford said services.

### Theme 3 social and emotional impacts: causes of stress and how they are overcome

#### Socialization and recreation

Some caregivers (15.1%) reported that they experienced a change in the type or amount of recreational, physical, religious, cultural, or social activities they engage in regularly. Before their child’s diagnosis, caregivers reported spending an average of 10.7 h (SD = 9.9) on leisure activities per week. However, after their child was diagnosed, this amount seems to have decreased to an average of 6.7 h per week (SD = 9.1). One caregiver also mentioned how they did not spend less time on leisure activities, although the types of activities they engaged in changed to better capture the interests of their child. Mixed responses were provided regarding how caregivers’ ability to socialize was affected. While most caregivers (57.5%) indicated that their socialization had been negatively impacted, 17.8% indicated that it had not been affected at all, and 24.6% reported that it had improved. These somewhat conflicting findings suggest that people’s reports of the impact of their child’s diagnosis on their socialization and recreation may, in part, be shaped by individual perspectives and attitudes (26). The improved socialization may also result from caregivers interacting with other caregivers of autistic children or increased participation in support groups.

#### Relationships with partners and other children

Caregiver reports were mixed regarding how their child’s autism diagnosis affected their relationship with their partner and/or their other children. Those who were negatively impacted in their relationships with their partner (39.7%) stated how the conflict that arose from co-parenting, paired with increasing caregiving demands and less time for self-care, led to high stress and emotional strain in relationships, which in turn caused relationship conflict. Similarly, 27.4% of caregivers felt that their relationship with their other children was negatively impacted due to increasing caregiving demands detracting from time spent attending to and spending time with their children without autism, as well as increased stress affecting their interactions overall. One caregiver summarized how all the different stressors they experience compound and affect their relationships:

Our child’s diagnosis has increased the practical demands on each parent, providing care as if for a toddler for the lifetime of our child. (We now have) limits on social activities, hobbies, and fitness due to financial limits, as well as physical exhaustion and lack of sleep. We conflict over parenting availability and our partnership role.

In contrast, people whose relationships with their partner were positively impacted (28.8%) said that their child receiving an autism diagnosis helped them better understand their child, allowing them to work more effectively as a team and bond over a shared understanding of their child’s behaviors. Some caregivers (38.4%) also said that their relationship with their other children had improved due to having a better understanding of each other’s needs and helping each other out with caregiving responsibilities. One caregiver elaborated that they still experience challenges within their relationships, but willingness to grow and having a positive outlook help to overcome them:

My child’s diagnosis has brought us closer in some ways as we have had to work as a team to support our child and navigate the challenges together. We’ve learned to communicate better, divide responsibilities, and celebrate the small victories along the way. Seeing how much we both care and want the best for our child has strengthened our bond, even if there are still challenges we face as a family.

#### Caregiver quality of life

Considering the financial, practical, social, and emotional challenges caregivers experience, it is understandable that almost all caregivers (97.3%) reported experiencing moderate to severe overall stress as a parent, with 74% reporting severe, very severe and worst possible stress. However, caregivers also cited a variety of sources that helped them alleviate this stress. Seventy-seven percent of participants (*N* = 57) reported receiving at least one form of social support. Among those receiving support, 44.6% (*N* = 33) reported receiving financial support, 24% (*N* = 18) received in-kind support, and 51% (*N* = 38) received emotional support. Family members (including parents, siblings, and extended family members) were the primary providers across all support types, accounting for 67% of financial support, 72% of in-kind support, and 76% of emotional support. Friends were also notable support providers, with 48% of participants receiving financial support, 50% receiving in-kind support, and 61% receiving emotional support from friends. Community support groups and other families with autistic children played an additional role. Thirty percent (15%) of participants reported receiving financial support, 28% (28%) reported receiving in-kind support, and 22% (29%) reported receiving emotional support from other families of autistic children (support groups). Furthermore, 81% of caregivers reported accessing at least one of the following services within their community: individual counseling, couples counseling, family therapy, and parent training. These types of support are particularly good at addressing relational and emotional challenges, improving mental health, and fostering a positive outlook on life. As one parent wrote within the feedback section of their survey:

Through parent training, we learned a lot about not seeing autism as a tragedy, but as an opportunity to rise to a level of growth that is needed to help the kids. We learned that happiness is a choice… this is an invaluable lesson for autism. It brings a lot of financial complication, but when parents are able to work on the happiness of the household, that really helps the journey.

Another source of support that was discussed during the focus group was online social media groups, which often consisted of people with similar lived experiences. These groups were beneficial to caregivers because they helped foster a sense of belonging, which is especially important for caregivers who spend less time in their community due to their increased work and/or caregiving demands.

## Discussion

This is the first study to our knowledge to provide comprehensive estimates of the economic impact of a childhood autism diagnosis in Ontario, Canada. In our pilot study, caregivers who have autistic children experienced impacts on their finances due to high out-of-pocket costs associated with diagnostic services, support services, and other indirect expenses including increased living expenses, childcare and education costs, and training costs. Families’ work-life balance was also affected due to changes in employment, schooling, housing, and caregiving. These impacts affected caregiver’s social lives and emotional well-being. However, whether they were impacted positively or negatively depended somewhat on the amount of support they received, including financial, social, and in-kind support.

Among out-of-pocket autism-related medical and therapy costs reported in our pilot sample, ABA Therapy had the highest expenses, with a median value of $1,000 and maximum costs reaching up to $16,000. These out-of-pocket expenses were incurred in addition to the funding provided by the OAP, which offers families up to $65,000 per year for core clinical services including ABA, depending on the child’s assessed needs (27). Ontario Association for Behavior Analysis’ (ONTABA) report, found the median hourly fee for in-person one-on-one services delivered by a Registered Behavior Analyst to be $130 (28). At these rates, families can only purchase a limited number of therapy hours beyond what is covered by public funding. While the current findings cannot be generalized to all families with autistic children in Ontario, they highlight how quickly families in our study can exhaust both government-provided and personal financial resources to access clinically recommended levels of care. Additionally, it should be noted that while our focus on families’ out-of-pocket costs aligns with this study’s goal of quantifying unmet financial needs for affected families and benefits from reduced recall bias because of their direct financial impacts on families, these estimates do not include expenses covered by third-party payers such as insurance or public programs. For example, OAP’s annual funding of up to $65,000 per child, as discussed above, is not reflected in the estimated costs. Therefore, the reported out-of-pocket costs should be considered as a lower bound of the total economic impacts on families.

Nevertheless, in our pilot sample, the already high out-of-pocket costs were much lower than the non-medical and non-therapy-related costs. Among the latter, participants most frequently reported increased costs for living expenses, childcare (both during school terms and holidays), and training, with median values ranging from $4,800 to $6,100 and maximum costs up to $432,000. This aligns with international estimates of the economic impact of autism (8, 9, 20), though our small sample preclude drawing generalizable conclusions about Ontario or Canada as a whole. Within our data, caregivers of children with higher support needs generally reported higher overall costs, however, financial hardship was reported across participants regardless of the level of their children’s needs. Many caregivers reported sacrificing their other expenses, going into debt, or relying financially on external sources to afford the costs of their child’s needs. While these findings highlight important challenges faced by families in our sample, they should be interpreted as exploratory evidence and indicate the need for future research using larger, stratified samples to improve our knowledge of the cost and cost-effectiveness of supports and services for autistic children. In doing so, this may provide a stronger foundation for supporting policy and decision-makers as they consider how best to allocate financial and practical supports for autistic children and their families. In addition, future research should compare the expenditures of families with and without autistic children to identify specific areas, such as education or productivity, that could be targeted via publicly provided supports.

It is also important to note the significant emotional cost related to the time factor experienced by caregivers in our pilot study due to their children being placed on waitlists for funding and support services. When children go an extended period without receiving crucial support services, particularly early intervention services, there is an increased likelihood of experiencing developmental consequences, including a lack of improvement in intellectual or adaptive functioning (29–31). This is especially true for children with more extensive support needs, as they require a greater variety of more intensive support services (32). Interestingly, our analysis of cost patterns based on support needs revealed a nonlinear relationship: families of children categorized as “requiring support for only a few aspects of life” reported higher spending than those in the “requires support for some aspects of life” group. As discussed in the Results section, this unexpected pattern may in part reflect small sample size, it may also occur as a result of tiered OAP funding where children are assigned based on an assessment of their support needs. Those who fall into lower or borderline tiers may receive less public support despite having needs that still require substantial financial investment from families. Therefore, in addition to suggesting complex relationship between support needs and costs, as noted in the Results section, this finding also underscores the limitations of relying on a single-item measure of support needs and the need for incorporating a more comprehensive measure, which we plan to implement in the full-scale survey with a larger sample size to enable more robust analysis.

Participants in our sample also described a potential opportunity cost associated with the time spent attempting to navigate informational resources on support services. Without accessible and clear information, caregivers may make ill-informed decisions, such as paying for a service that is not beneficial to their child or remaining on waitlists for services they do not need resulting in financial and time-related losses. However, the challenge is not only about information availability but also about the design of the service system itself. Previous research has shown that when families are made responsible for coordinating services without adequate support or navigation resources the system becomes overwhelming and burdensome (33). As such, families may find themselves acting as unpaid case managers, navigating opaque systems. Even though organizations like Autism Ontario provide information, caregivers in our study still faced difficulty finding what they needed, underscoring the importance of policy interventions that streamline navigation supports and actively engage families in service design and delivery.

Moreover, caregivers in our sample also emphasized the importance of advocating for their right to affordable and accessible support services, both for their children and for themselves. Consistent with previous research, caregivers in this study experienced various challenges relating to work, school, and caregiving, some of which had indirect financial impacts, including loss of employment or productivity, inadequate support from schools, and designating caregiving responsibilities (19, 23, 34). All the caregivers in this study also sought some form of publicly or privately provided support services, such as therapy or counseling, which several reported alleviated the impact of the aforementioned challenges on their well-being. While it would be beneficial for policies to be in place that ensure the affordability and accessibility of these services to caregivers, sufficient support and accommodations to caregivers within work and school, may reduce caregiver stress that results in the need for such services.

The above-discussed challenges reported by participants in our pilot study align with broader concerns about access to autism services and the economic impacts on families worldwide. Notably, such challenges are often experienced on an even larger scale in low- and middle-income countries (LMICs), where the economic struggles faced by caregivers are exacerbated by a lack of support from governments and other community organizations (35). For example, in Ethiopia, autism-specific services are extremely limited and are almost exclusively offered in the country’s capital city, resulting in extremely long waitlists (36). Limited knowledge regarding the aetiology and characteristics of autism also makes training personnel to diagnose and treat the condition difficult (37). Therefore, although the economic impacts of caring for autistic children are experienced globally, it is essential to consider political, economic, and social contexts when identifying solutions. While framing our pilot findings within this global context underscores their public health relevance, it must also be acknowledged that the results are exploratory and cannot be generalized beyond our sample.

Finally, as mentioned by one caregiver in this study, it is important to remember that there is more to an autism diagnosis than increased financial, practical, and social burdens. Rather than emphasizing the stressors associated with having an autistic child, focusing on the more positive aspects of caregiving can help improve caregivers’ well-being by building resilience. Celebrating children’s developmental milestones, spending quality time with one’s child, and gaining a better understanding of their child through diagnosis, are all more positive ways one can view their familial experience (38). Service providers can capitalize on this by promoting the positives of caregiving, highlighting the strengths of caregivers of autistic children, and drawing attention to the positive contributions autistic children can make to their families, potentially enabling parents to cope more successfully with challenges that arise (39).

### Quality of the current survey instrument

Within the pilot survey, we included a section asking participants for feedback on the survey design. The vast majority of participants (96%) reported that all questions in the survey instrument’s current version were clear and easy to follow. However, we also received comments on ways to improve this survey and suggestions for including additional types of costs to refine the survey. For example, one participant mentioned that while her current family composition is a two-parent household, it does not include her son’s father. As a result, some information may be missing since the current pilot survey only asked about the employment and education information of the current partner, who may differ from the child’s biological parent. Only 4% (*N* = 3) of participants reported either a divorce or a separated status in the pilot survey. However, this might impact the full-scale survey with a broader participant base. We will revise and add relevant questions in the full-scale survey.

Additionally, we identified areas for improvement during the data analysis process. For example, when asking for the medical and therapy costs, we focused on the out-of-pocket payment by the caregivers because these are the costs that caregivers may have the most vivid memory. However, these out-of-pocket costs do not necessarily reflect the actual autism-related medical and therapy costs. In some cases, costs are fully or partially covered by the government or private insurance. Therefore, the true autism-related medical and therapy costs are likely to be higher than the reported out-of-pocket costs, and it is important to capture such information to help better understand its overall social costs. Therefore, we will refine the current survey version to include such questions in the full-scale survey.

We acknowledge that the small sample size of this pilot survey limits the extent to which the findings can be generalized. In the full-scale survey, we aim to address this limitation by adopting a broader sampling strategy with particular efforts to ensure adequate representation of families from rural and socioeconomically disadvantaged backgrounds (e.g., newcomer and Indigenous families who may face disproportionate barriers to accessing services). Recruitment will be supported through partnerships with community organizations and social service providers that work closely with these families, to help ensure that future findings capture a wider range of family experiences and strengthen their policy relevance.

## Conclusion

The purpose of this pilot study was to test and refine a survey instrument aimed at reaching a broader population to gain a more comprehensive understanding of the economic impacts of children’s autism diagnosis on their families. This study is the first step in a series of investigations examining how families with autistic children respond to economic costs caused by the diagnoses of their children’s ASD in Canada. While limited in their generalizability, the results reported in this study offer important context and direction for future, larger-scale research and policy development. The next step will be to launch a full-scale survey to a wider number of participants so that we can conduct robust quantitative analysis using econometrics models and provide a more comprehensive picture of the economic impact of a child’s autism diagnosis on affected families and communities.

## Data Availability

The raw data supporting the conclusions of this article will be made available by the authors, without undue reservation.

## Footnotes

^1^Valid responses are defined as those that are logically consistent; only one response was excluded due to logical inconsistent.

^2^See the report by Autism Ontario: https://www.autismontario.com/news/autism-ontario-responds-ministry-children-community-and-social-services. It should be noted that this report was written in 2019, and the wait list has grown even longer by 2025 (the year this study is conducted).

^3^Although financial support for these private assessments is available in Ontario through programs such as the Ontario Autism Program (OAP), families again face a long waitlist to join the program, and therefore to receive such financial support in a timely manner. For example, it is reported that, in 2024, there are 50,000 children waiting to be enrolled into OAP (Source: https://opencouncil.ca/ontario-autism-program/).

## References

[1] American Psychiatric Association ed. Diagnostic and statistical manual of mental disorders. 5th ed. Washington, DC: American Psychiatric Association (2022). 2022 p.

[2] Statistics Canada. (2019). Canadian health survey on children and youth (CHSCY). Ottawa, ON. Available online at: https://www150.statcan.gc.ca/n1/daily-quotidien/200723/dq200723a-eng.htm

[3] Birnbaum R Lach L Saposnek D. Children with neurodevelopmental disorders in parental separation and divorce In: Drozd L Saini M Olesen N, editors. Parenting plan evaluations: Applied research for the family court. 2nd ed. New York: Oxford University Press (2016). 205–43.

[4] Cadman T Eklund H Howley D Hayward H Clarke H Findon J. Caregiver burden as people with autism spectrum disorder and attention-deficit/hyperactivity disorder transition into adolescence and adulthood in the United Kingdom. J Am Acad Child Adolesc Psychiatry. (2012) 51:879–88. doi: 10.1016/j.jaac.2012.06.017, PMID: 22917201

[5] Misquiatti ARN Brito MC Ferreira FTS Assumpção Júnior FB. Family burden and children with autism spectrum disorders: perspective of caregivers. Rev CEFAC. (2015) 17:192–200. doi: 10.1590/1982-0216201520413

[6] Stuart M McGrew JH. Caregiver burden after receiving a diagnosis of an autism spectrum disorder. Res Autism Spectr Disord. (2009) 3:86–97. doi: 10.1016/j.rasd.2008.04.006

[7] Zablotsky B Bradshaw CP Stuart EA. The association between mental health, stress, and coping supports in mothers of children with autism spectrum disorders. J Autism Dev Disord. (2013) 43:1380–93. doi: 10.1007/s10803-012-1693-7, PMID: 23100053

[8] Lavelle TA Weinstein MC Newhouse JP Munir K Kuhlthau KA Prosser LA. Economic burden of childhood autism spectrum disorders. Pediatrics. (2014) 133:e520–9. doi: 10.1542/peds.2013-0763, PMID: 24515505 PMC7034397

[9] Liao X Li Y. Economic burdens on parents of children with autism: a literature review. CNS Spectr. (2020) 25:468–74. doi: 10.1017/S1092852919001512, PMID: 31709959

[10] de Oliveira C Tanner B. The health care costs of caring for children and adolescents with autism spectrum disorder: a population-based analysis. Res Autism Spectr Disord. (2023) 108:102255. doi: 10.1016/j.rasd.2023.102255

[11] Baber R. (2019). RE: Ontario autism plan. Available online at:https://drive.google.com/file/d/1GI4DxxFyZOuFn33mLNXaWyaDnla1XWxj/view

[12] Montes G Halterman JS. Association of childhood autism spectrum disorders and loss of family income. Pediatrics. (2008) 121:e821–6. doi: 10.1542/peds.2007-1594, PMID: 18381511

[13] Dudley C Emery JC. The value of caregiver time: costs of support and care for individuals living with autism spectrum disorder. SPP Research Paper. (2014). doi: 10.2139/ssrn.2379633

[14] McLaughlin J. Schneider M.. (2019). Autism services in Ontario: Impacts on family and child well-being, research summary. Waterloo, ON: Laurier Autism Research Consortium. Available online at: https://www.wlu.ca/academics/faculties/faculty-of-human-and-social-sciences/faculty-profiles/janet-mclaughlin/larc/assets/documents/larc-report.pdf

[15] Buescher AV Cidav Z Knapp M Mandell DS. Costs of autism spectrum disorders in the United Kingdom and the United States. JAMA Pediatr. (2014) 168:721–8. doi: 10.1001/jamapediatrics.2014.210, PMID: 24911948

[16] Ganz ML. The lifetime distribution of the incremental societal costs of autism. Arch Pediatr Adolesc Med. (2007) 161:343–9. doi: 10.1001/archpedi.161.4.343, PMID: 17404130

[17] Järbrink K Fombonne E Knapp M. Measuring the parental, service and cost impacts of children with autistic spectrum disorder: a pilot study. J Autism Dev Disord. (2003) 33:395–402. doi: 10.1023/A:1025058711465, PMID: 12959418

[18] Järbrink K. The economic consequences of autistic spectrum disorder among children in a Swedish municipality. Autism. (2007) 11:453–63. doi: 10.1177/1362361307079602, PMID: 17942458

[19] Knapp M Romeo R Beecham J. Economic cost of autism in the UK. Autism. (2009) 13:317–36. doi: 10.1177/1362361309104246, PMID: 19369391

[20] Roddy A O'Neill C. The economic costs and its predictors for childhood autism spectrum disorders in Ireland: how is the burden distributed? Autism. (2019) 23:1106–18. doi: 10.1177/1362361318801586, PMID: 30270653

[21] Perry A Taheri A Ting V Weiss J. The GO4KIDDS brief adaptive scale. J Appl Res Intellect Disabil. (2015) 28:594–7. doi: 10.1111/jar.12143, PMID: 25846705

[22] Vaismoradi M Turunen H Bondas T. Content analysis and thematic analysis: implications for conducting a qualitative descriptive study. Nurs Health Sci. (2013) 15:398–405. doi: 10.1111/nhs.12048, PMID: 23480423

[23] Rogge N Janssen J. The economic costs of autism spectrum disorder: a literature review. J Autism Dev Disord. (2019) 49:2873–900. doi: 10.1007/s10803-019-04014-z, PMID: 30976961

[24] Burton P Chen K Lethbridge L Phipps S. Child health and parental paid work. Rev Econ Househ. (2017) 15:597–620. doi: 10.1007/s11150-014-9251-z

[25] Lynch FL Bulkley JE Varga A Crawford P Croen LA Daida YG. The impact of autism spectrum disorder on parent employment: results from the r-kids study. Autism Res. (2023) 16:642–52. doi: 10.1002/aur.2882 36546608

[26] Runge RA Soellner R. Cultural bias in parent reports: the role of socialization goals when parents report on their child’s problem behavior. Child Psychiatry Hum Dev. (2024) 55:1020–30. doi: 10.1007/s10578-022-01464-y, PMID: 36371526 PMC11245439

[27] Ontario Ministry of Children, Community and Social Services (MCCSS) (2024) Ontario Autism Program: Guidelines for core clinical services and supports Available online at: https://www.ontario.ca/page/ontario-autism-program-guidelines-core-clinical-services-and-supports

[28] Ontario Association for Behaviour Analysis (2025) Insurance information report Available online at: https://ontaba.org

[29] Dimian AF Symons FJ Wolff JJ. Delay to early intensive behavioral intervention and educational outcomes for a Medicaid-enrolled cohort of children with autism. J Autism Dev Disord. (2021) 51:1054–66. doi: 10.1007/s10803-020-04586-1, PMID: 32642958 PMC12599662

[30] Flanagan HE Perry A Freeman NL. Effectiveness of large-scale community based intensive behavioral intervention: a waitlist comparison study exploring outcomes and predictors. Res Autism Spectr Disord. (2012) 6:673–82. doi: 10.1016/j.rasd.2011.09.011

[31] Smith T Klorman R Mruzek DW. Predicting outcome of community-based early intensive behavioral intervention for children with autism. J Abnorm Child Psychol. (2015) 43:1271–82. doi: 10.1007/s10802-015-0002-2, PMID: 25778537

[32] Brookman-Frazee L Baker-Ericzén M Stahmer A Mandell D Haine RA Hough RL. Involvement of youths with autism spectrum disorders or intellectual disabilities in multiple public service systems. J Ment Health Res Intellect Disabil. (2009) 2:201–19. doi: 10.1080/19315860902741542, PMID: 19809531 PMC2757308

[33] Gardiner E Iarocci G. Examining family quality of life within the context of a participant-directed ASD funding program in British Columbia, Canada. J Policy Pract Intellect Disabil. (2018) 15:110–23. doi: 10.1111/jppi.12237

[34] Hedley D Uljarević M Cameron L Halder S Richdale A Dissanayake C. Employment programmes and interventions targeting adults with autism spectrum disorder: a systematic review of the literature. Autism. (2017) 21:929–41. doi: 10.1177/1362361316661855, PMID: 27542395

[35] Kakooza-Mwesige A Bakare M Gaddour N Juneja M. The need to improve autism services in lower-resource settings. Lancet. (2022) 399:217–20. doi: 10.1016/S0140-6736(21)02658-1, PMID: 34883050

[36] Tekola B Baheretibeb Y Roth I Tilahun D Fekadu A Hanlon C. Challenges and opportunities to improve autism services in low-income countries: lessons from a situational analysis in Ethiopia. Global Mental Health. (2016) 3:e21. doi: 10.1017/gmh.2016.17, PMID: 28596889 PMC5454792

[37] Ruparelia K Abubakar A Badoe E Bakare M Visser K Chugani DC. Autism spectrum disorders in Africa: current challenges in identification, assessment, and treatment: a report on the international child neurology association meeting on ASD in Africa, Ghana, April 3-5, 2014. J Child Neurol. (2016) 31:1018–26. doi: 10.1177/0883073816635748, PMID: 26979098 PMC6858866

[38] Corman MK. The positives of caregiving: mothers' experiences caregiving for a child with autism. Fam Soc. (2009) 90:439–45. doi: 10.1606/1044-3894.3923

[39] Pierpont J. Emphasizing caregiver strengths to avoid out-of-home placement of children with severe emotional and behavioral disturbances. J Hum Behav Soc Environ. (2004) 9:5–17. doi: 10.1300/J137v09n01_02

